# The role of soluble fiber intake in patients under highly effective lipid-lowering therapy

**DOI:** 10.1186/1475-2891-10-80

**Published:** 2011-08-02

**Authors:** Silvia C Ramos, Francisco A Fonseca, Soraia H Kasmas, Flávio T Moreira, Tatiana Helfenstein, Ney C Borges, Ronilson A Moreno, Vinicius M Rezende, Fernanda C Silva, Maria C Izar

**Affiliations:** 1 Department of Medicine, Cardiology Division, Federal University of Sao Paulo, Rua Pedro de Toledo, 276, São Paulo, SP, 04039-030, Brazil; 2 Synchrophar, Praça Vinte e Oito de Fevereiro, 55, Paulínia, 13140-000, SP, Brazil

**Keywords:** Soluble fiber, lipids, campesterol, desmosterol, lipid-lowering therapy

## Abstract

**Background:**

It has been demonstrated that statins can increase intestinal sterol absorption. Augments in phytosterolemia seems related to cardiovascular disease.

**Objective:**

We examined the role of soluble fiber intake in endogenous cholesterol synthesis and in sterol absorption among subjects under highly effective lipid-lowering therapy.

**Design:**

In an open label, randomized, parallel-design study with blinded endpoints, subjects with primary hypercholesterolemia (n = 116) were assigned to receive during 12 weeks, a daily dose of 25 g of fiber (corresponding to 6 g of soluble fibers) plus rosuvastatin 40 mg (n = 28), rosuvastatin 40 mg alone (n = 30), sinvastatin 40 mg plus ezetimibe 10 mg plus 25 g of fiber (n = 28), or sinvastatin 40 mg plus ezetimibe 10 mg (n = 30) alone.

**Results:**

The four assigned therapies produced similar changes in total cholesterol, LDL-cholesterol, and triglycerides (p < 0.001 vs. baseline) and did not change HDL-cholesterol. Fiber intake decreased plasma campesterol (p < 0.001 vs. baseline), particularly among those patients receiving ezetimibe (p < 0.05 vs. other groups), and β-sitosterol (p = 0.03 vs. baseline), with a trend for lower levels in the group receiving fiber plus ezetimibe (p = 0.07). Treatment with rosuvastatin alone or combined with soluble fiber was associated with decreased levels of desmosterol (p = 0.003 vs. other groups). Compared to non-fiber supplemented individuals, those treated with fibers had weight loss (p = 0.04), reduced body mass index (p = 0.002) and blood glucose (p = 0.047).

**Conclusion:**

Among subjects treated with highly effective lipid-lowering therapy, the intake of 25 g of fibers added favorable effects, mainly by reducing phytosterolemia. Additional benefits include improvement in blood glucose and anthropometric parameters.

## Introduction

Dietary fiber is widely prescribed [1], alone or associated with lipid-lowering therapies, in order to reduce cholesterol levels [2]. The exact mechanism by which soluble fibers lower serum LDL-cholesterol levels is not completely understood. However, there are evidences suggesting that soluble fibers may interfere with lipid and/or bile acid metabolism [3].

Atherosclerosis has been recognized as a complex disease related in part to lipid disorders. Beyond cholesterol content of lipoproteins, a moderate increase in phytosterolemia seems related to cardiovascular disease [4,5].

Based on clinical trials, high doses of statins have been recommended to achieve lower levels of LDL-cholesterol [6-8]. Nevertheless, the use of high doses of statins is not always well tolerated or effective and the concomitant use of ezetimibe has been proposed. Due to the blockade of the endogenous cholesterol synthesis [9], statins appear related to increased intestinal absorption of sterols, both cholesterol and plant sterols [10,11]. Ezetimibe has an important synergism with statins in reducing LDL-cholesterol and is able to prevent the increase in intestinal sterols absorption [12,13]. On the other hand, the inhibition of cholesterol absorption increases the endogenous cholesterol synthesis [14-16].

The Framingham Offspring Study showed that cholesterol synthesis markers were associated with reduction in cardiovascular disease risk and, in contrast, absorption markers were associated with an almost two-fold increased risk [17].

Although changes in lifestyle, including a prudent diet [1] have been widely recommended for primary or secondary prevention of cardiovascular disease, the usefulness of a soluble fiber-enriched diet, in patients under highly effective lipid-lowering therapy is less reported, not only for the achievement of lipid goals, but particularly to the balance between phytosterolemia and cholesterol synthesis.

Therefore, we hypothesized that soluble fiber intake can reduce plant sterols absorption among subjects receiving highly effective lipid-lowering therapy. The role of fiber intake was tested in two different lipid-lowering strategies, using high-dose statin or the combination of a statin plus a cholesterol absorption inhibitor. These drugs and dosages were chosen to attain similar changes in lipid profile through distinct mechanisms.

## Subjects and methods

### Design and study population

We performed a prospective, randomized, open label study, with parallel arms and blinded endpoints. Patients were recruited from the outpatient unit of dyslipidemias of our university. The trial protocol was conducted in accordance with the ethical standards of the institution on human experimentation and approval was obtained from the local ethics committee. Informed consent was obtained from all participants prior inclusion. Eligible patients were men and women, 30 to 75 years of age, in primary or secondary prevention of coronary heart disease, who had an indication for lipid-lowering therapy in accordance with the National Cholesterol Education Program/Adult Treatment Panel (NCEP/ATP III) guidelines [1]. A total of 116 subjects completed the study protocol. Patients with liver, renal or gastrointestinal disease, malignancies, uncontrolled metabolic disorder, that might affect the tolerability or safety of the treatments were excluded. Exclusion criteria during the study were low adherence (less than 80%) to either the lipid-lowering regimen or to the daily fiber intake. The major characteristics of the study population are listed in the Table 1. Risk factors and metabolic syndrome were defined by the NCEP/ATP III guidelines [1].

**Table 1:** Baseline characteristics of the study population by group

	RSV+Fib	RSV	SIM/EZE+Fib	SIM+EZE	P-value
	N = 28	N = 30	N = 28	n = 30	
Age, years^1^	57 (1)	60 (2)	62 (1)	58 (1)	0.21
Female, n (%)^2^	21 (75)	20 (67)	19 (68)	22 (73)	0.87
Hypertension, n (%)^2^	22 (79)	24 (80)	18 (64)	26 (87)	0.22
Diabetes, n (%)^2^	5 (18)	7 (23)	6 (21)	9 (30)	0.73
Smoking, n (%)^2^	2 (7)	3 (10)	1 (4)	2 (7)	0.82
Metabolic Syndrome, n (%)^2^	17 (61)	18 (60)	13 (46)	14 (47)	0.53

Differences between groups were analyzed by ANOVA^1^, or Pearson's chi-square test^2^. RSV = rosuvastatin; Fib = fiber; SIM = simvastatin; EZE = ezetimibe.

The 24-hour dietary recall [18] was obtained at the beginning and end of the study. Before treatment, all patients received nutritional counseling based on the Therapeutic Lifestyle Changes of the NCEP/ATP III [1]. Then, they were randomized to receive or not 44 g of the passion fruit peel flour, to ensure a minimum daily consumption of 6 g of soluble fiber and to achieve the target of 25 g of fiber intake, divided into three daily doses administered before meals. They were also randomized to rosuvastatin 40 mg or the combination of simvastatin 40 mg plus ezetimibe 10 mg, daily for 12 weeks. The lipid-lowering agents and the fiber were given to the patients every 30 days, with reinforcement of lifestyle changes and to evaluate the adherence to the study protocol.

### Study drugs and fiber

Rosuvastatin (Crestor ^®^, IPR Pharmaceuticals, Porto Rico), Simvastatin/Ezetimibe (Zetsim^®^, Schering-Plough Products, Las Piedras, Porto Rico) were gifts from AstraZeneca and Merck Co, respectively. The passion fruit peel flour was purchased from Tango alimentos (Londrina, PR, Brazil). The composition of passion fruit peel flour was analyzed by Centro de Ciências e Qualidade de Alimentos (Instituto de Tecnologia de Alimentos, Campinas, SP, Brazil), which revealed that 44 g of flour corresponded to 45 kcal of total energy, being 25 g of total fiber, 6 g of soluble fiber, 5 mg of campesterol, and 35 mg of β-sitosterol.

### Blood sample collection and assays

#### Lipids and biochemistry

Biochemical analyses were performed in samples obtained after a 12-hour fasting period at baseline and after 12 weeks of treatment in a central laboratory of our university using automated techniques (Advia 2400, Siemens Healthcare Diagnostics, Tokyo, Japan). Serum cholesterol, HDL-cholesterol, and triglycerides were determined by automated methods (Advia 2400, Siemens Healthcare Diagnostics, Tokyo, Japan). LDL-cholesterol was calculated using the Friedewald formula [19]. Glycated hemoglobin was assayed by high-performance liquid chromatopraphy (Tosho G2, Tosho Inc., Tokyo, Japan), apolipoprotein A1, apolipoprotein B, and highly-sensitive C-reactive protein were determined by nephelometry (Array 360 CE/AL, Beckmann Coulter, Inc. Brea, CA).

#### Phytosterols and desmosterol

For the quantification of beta-sitosterol and campesterol (markers of sterols absorption), as well as for desmosterol (precursor of the endogenous cholesterol synthesis) we used ultra performance liquid chromatography (UPLC) and mass spectrometry (MS). Briefly, these sterols were quantitated in plasma samples by a method developed and run by Synchrophar, Campinas, SP, Brazil. The sterols were detected as its free forms, i.e., non-esterified, monitoring the ions with m/z, 367.30 for desmosterol, 397.25 for β-sitosterol and 383.60 for campesterol. The levels of compounds were determined by comparison of peak response against a calibration curve from 0.5 μg/mL to 10.0 μg/mL. Samples presenting higher levels than 10.0 μg/mL were diluted to compare with calibration levels. Results were transformed to mg/dL.

### Statistical analyses

Results are expressed as mean (SEM) or percentages unless otherwise specified. Continuous variables were tested for distribution of normality by Kolmogorov-Smirnov test. Comparisons between groups at baseline were made by ANOVA or Pearson's Chi square test. For comparisons between timepoints and groups we used General Linear Model (GLM) - repeated measures or Kruskal-Wallis test, when data were presented as percentages. To compare non-fiber supplemented and fiber-supplemented groups, the 2-sided Student's independent or paired t-test or Mann-Whitney test were used. When appropriate, continuous variables were log transformed. Statistical significance was set at a p-value < 0.05. All analyses were made using the SPSS 17.0 for windows (SPSS Inc, Chicago, IL).

## Results

### Dietary intake, weight and body mass index

The analyses of the dietary recall have shown that the consumption of cholesterol and dietary fiber did not differ between groups; monounsaturated, polyunsaturated, or *trans-*fatty acids were also similar. Energy intake was reduced at the 12 weeks (p = 0.001 vs. baseline, GLM-repeated measures), with energy from carbohydrates, fatty acids, and proteins being comparable among groups. The estimated daily fiber intakes, obtained from dietary recall were not different along the study, when fiber supplementation with passion fruit peel flour was not counted (Table 2).

**Table 2:** Characteristics of the diet consumed at baseline and 12 weeks, by group

	RSV+Fib	RSV	SIM/EZE+Fib	SIM+EZE	P-value
	N = 28	N = 30	N = 28	n = 30	
MUFA%^1^					
Baseline	13.8 (0.7)	12.8 (0.7)	13.4 (1.0)	11.8 (0.7)	0.374
12 weeks	12.9 (0.6)	13.6 (0.8)	13.4 (0.5)	11.5 (0.6)	0.094
PUFA%^1^					
Baseline	9.1 (0.6)	11.9 (1.0)	10.3 (0.8)	10.3 (1.0)	0.329
12 weeks	8.3 (0.8)	10.7 (1.1)	9.6 (0.6)	8.2 (0.7)	0.098
SAFA%^1^					
Baseline	9.7 (0.6)	9.5 (0.6)	9.5 (0.5)	9.1 (0.6)	0.833
12 weeks	9.3 (0.5)	9.4 (0.5)	9.5 (0.7)	8.9 (0.7)	0.717
*Trans *FA%^1^					
Baseline	0.3 (0.1)	0.4 (0.1)	0.3 (0.1)	0.3 (0.1)	0.608
12 weeks	0.3 (0.1)	0.4 (0.1)	0.4 (0.2)	0.4 (0.1)	0.237
Energy Kcal/day^2^					
Baseline	1762 (116)	1766 (100)	1828 (156)	1741 (136)	0.001^a^
12 weeks	1653 (116)	1520 (85)	1585 (129)	1599 (102)	0.990^b^
Lipids%^1^					
Baseline	36.1 (1.4)	37.3 (1.9)	36.3 (1.7)	34.3 (1.8)	0.941
12 weeks	33.9 (1.4)	36.9 (1.8)	35.7 (1.2)	31.4 (1.6)	0.041
Carbohydrates%^1^					
Baseline	46.4 (1.9)	46.9 (2.1)	46.7 (2.3)	49.2 (2.1)	0.869
12 weeks	46.3 (1.5)	46.5 (1.6)	46.7 (1.7)	52.1 (1.8)	0.034
Proteins%^1^					
Baseline	18 (1.3)	16.5 (1.0)	17.7 (1.3)	17.3 (0.9)	0.79
12 weeks	20 (1.0)	17.6 (0.9)	18.5 (1.2)	17.8 (0.8)	0.268
Cholesterol mg/day^2^					
Baseline	214 (29)	200 (21)	197 (33)	210 (29)	0.203^a^
12 weeks	222 (29)	157 (12)	201 (34)	171 (22)	0.683^b^
Fibers g/day^2^					
Baseline	20 (3)	17 (1)	22 (3)	16 (2)	0.853^a^
12 weeks	20 (2)	16 (1)	18 (2)	19 (2)	0.666^a^

Differences analyzed by Kruskal-Wallis test^1 ^or General Linear Model-repeated measures followed by Tukey's test^2^, with p-values for comparisons ^a^between visits and ^b^between groups.

MUFA = monounsaturated fatty acids; PUFA = polyunsaturated fatty acids; SAFA = saturated fatty acids; RSV = rosuvastatin 40 mg; Fib = fiber; SIM = simvastatin 40 mg; EZE = ezetimibe 10 mg; FA = fatty acids.

**Figure 1:**
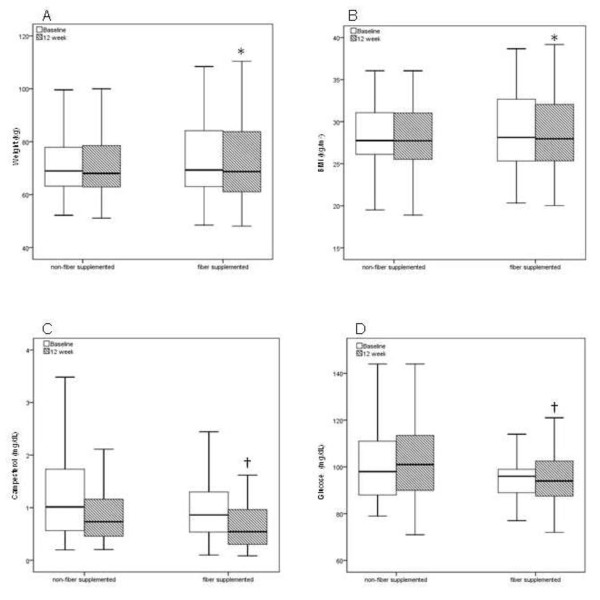
**Box plots of (A) Weight, (B) BMI, (C) Campesterol and (D) Glucose at baseline (white bars) and 12 weeks (dashed bars)**. Fiber-supplemented subjects presented mild reduction in body weight (*p = 0.04 vs. non-fiber supplemented, Student's paired t-test), body mass index (*p = 0.002 vs. non-fiber supplemented, Student's paired t-test), reduction in campesterol levels (*p = 0.033 vs. non-fiber supplemented, Student's paired t-test; †p = 0.025 vs. non-fiber supplemented, Student's independent t-test) and in blood glucose (†p = 0.035 vs. non-fiber supplemented, Student's independent t-test). BMI = body mass index.

Regarding the soluble fiber status, those with the addition of fiber presented weight (p = 0.04, Student's t-test) and BMI reduction (p = 0.002, Student's t-test) on week 12 (Figure 1A-B).

### Laboratory results

#### Lipids and apolipoproteins

In Table 3 we present our main laboratory findings. Twelve-week treatment with lipid-lowering agents with or without fiber supplementation was associated with effective reduction of total cholesterol (p < 0.0001 vs. baseline, GLM-repeated measures), LDL-cholesterol (p < 0.001 vs. baseline, GLM-repeated measures) and triglycerides (p < 0.001 vs. baseline, GLM-repeated measures), without changes in HDL-cholesterol levels. Apolipoprotein B serum levels were lower after a 12-week treatment period (p < 0.001 vs. baseline, GLM-repeated measures), whereas we observed higher levels of Apolipoprotein A1 (p = 0.035 vs. baseline, GLM-repeated measures), without differences between treatments. Differences on lipids and apolipoproteins according to fiber intake status were not significant (data not shown).

**Table 3:** Anthropometric and laboratory parameters at baseline and 12 weeks, by group

	RSV+Fib	RSV	SIM/EZE+Fib	SIM+EZE	P-value^1^	P-value^2^
	N = 28	N = 30	N = 28	n = 30		
Weight kg						
Baseline	74 (3)	72 (2)	73 (3)	70 (2)	0.001	0.803
12 weeks	73 (3)	72 (3)	72 (3)	70 (2)		
BMI kg/m^2^						
Baseline	28 (1)	30 (1)	30 (1)	28 (1)	0.005	0.246
12 weeks	27 (1)	30 (1)	29 (1)	28 (1)		
Glucose mg/dL						
Baseline	97 (2)	104 (4)	103 (5)	108 (7)	0.754	0.257
12 weeks	95 (2)	104 (4)	100 (4)	109 (6)		
HbA1c %						
Baseline	5.7 (0.1)	6.3 (0.2)	6.0 (0.2)	6.2 (0.2)	0.02	0.215
12 weeks	5.8 (0.1)	6.2 (0.2)	6.1 (0.2)	6.3 (0.2)		
hsCRP mg/L						
Baseline	4.2 (1.1)	3.7 (0.5)	3.3 (0.4)	4.5 (1.3)	<0.001	0.976
12 weeks	2.4 (0.5)	2.5 (0.4)	2.3 (0.4)	2.3 (0.4)		
Cholesterol mg/dL						
Baseline	256 (10)	244 (8)	247 (8)	241 (9)	<0.0001	0.639
12 weeks	149 (6)	140 (5)	155 (9)	147 (8)		
LDL-C mg/dL						
Baseline	167 (8)	154 (7)	162 (6)	158 (8)	<0.001	0.436
12 weeks	70 (5)	64 (4)	80 (8)	72 (4)		
HDL-C mg/dL						
Baseline	57 (3)	52 (2)	52 (2)	54 (2)	0.392	0.351
12 weeks	57 (3)	50 (3)	53 (3)	52 (2)		
Triglycerides mg/dL						
Baseline	160 (13)	187 (16)	158 (14)	144 (10)	<0.001	0.291
12 weeks	110 (9)	123 (8)	110 (7)	108 (9)		
Apoliprotein A1 mg/dL						
Baseline	153 (6)	145 (6)	141 (4)	147 (3)	0.035	0.482
12 weeks	155 (6)	149 (6)	145 (5)	152 (4)		
Apoliprotein B mg/dL						
Baseline	133 (6)	133 (5)	133 (5)	134 (6)	<0.001	0.775
12 weeks	68 (3)	67 (3)	73 (5)	70 (4)		
Campesterol mg/dL						
Baseline	1.2 (0.2)	1.1 (0.1)	0.9 (0.1)	1.3 (0.2)	<0.001	0.005
12 weeks	0.9 (0.1)	1.0 (0.1)	0.5 (0.1)	0.8 (0.1)		
β-sitosterol mg/dL						
Baseline	0.7 (0.1)	0.8 (0.1)	0.6 (0.1)	0.9 (0.1)	0.03	0.072
12 weeks	0.6 (0.6)	0.7 (0.1)	0.5 (0.1)	0.6 (0.1)		
Desmosterol mg/dL						
Baseline	0.7 (0.1)	1.9 (1.6)	0.9 (0.3)	0.5 (0.1)	0.005	0.373
12 weeks	0.6 (0.1)	0.9 (0.5)	1.0 (0.3)	1.6 (0.5)		

Differences analyzed by General Linear Model-repeated measures, followed by Tukey's test. HbA1c = glycated hemoglobin; hs-CRP = highly-sensitive C-reactive protein; RSV = rosuvastatin 40 mg; Fib = fiber; SIM = simvastatin 40 mg; EZE = ezetimibe 10 mg. ^1^Differences between baseline and 12 week; ^2^Differences between groups.

#### Desmosterol and phytosterols

Desmosterol plasma levels presented interaction between groups. Subjects receiving rosuvastatin have shown decreased levels of desmosterol in comparison with the subjects treated with simvastatin plus ezetimibe (p = 0.003 vs. other groups)

Sterol intestine absorption markers, campesterol and β-sitosterol, are presented in Table 3. There was a decrease in campesterol plasma levels at the end of treatment (p < 0.001 vs. baseline, GLM-repeated measures), with lower levels observed in subjects receiving fibers and treated with simvastatin plus ezetimibe (p = 0.005 between groups, GLM-repeated measures). Campesterol levels were lower for those subjects taking soluble fibers on week 12 (p = 0.025 vs. non-fiber supplemented, Student's independent t-test) as shown in Figure 1C. There was a decrease in plasma levels of β-sitosterol (p = 0.03 vs. baseline) with a trend for lower levels in the group receiving fibers plus ezetimibe (p = 0.07). Fiber intake status did not affect β-sitosterol plasma levels.

#### Glucose, glycated hemoglobin and C-reactive protein

Blood glucose and glycated hemoglobin (HbA1c) did not change along the study between groups (Table 3). However, there were differences in glucose percent change in subjects supplemented with fibers when compared with those not receiving fibers [mean (SEM) - 2.9 (1.8) % vs. 0.6 (4.3) %, p = 0.038, Mann-Whitney test]. Glucose levels were lower in subjects supplemented with fibers on week 12 (p = 0.035 vs. non-supplemented, Student's independent t-test); data shown in Figure 1D.

Highly-sensitive C-reactive protein serum levels were elevated at baseline and decreased similarly between treatment groups (p < 0.001 vs. baseline, GLM-repeated measures) (Table 3). We did not observe differences in highly-sensitive C-reactive protein according to fiber intake status (data not shown).

## Discussion

This study examined the role of fiber supplementation in patients under effective therapy with lipid-lowering drugs. It has been reported that consumption of soluble fibers promotes a moderate effect in lowering cholesterol in hypercholesterolemic patients [20,21]. However, the literature is scarce in relation to the benefit of fibers added to therapy in patients taking effective lipid-lowering agents. Our study has shown that no further reduction was achieved in total cholesterol, LDL-cholesterol, and triglycerides when fibers were added to an effective therapy. However, fibers seemed to act synergistically with ezetimibe, reducing phytosterolemia, at the recommended dose for total (25 g) and soluble fibers (6 g) intake [1].

However, the addition of fibers to the diet, even in patients receiving highly effective therapy can bring important benefits [22,23]. It has been reported that use of statins alone may increase the absorption of sterols by the intestine, causing mild to moderate increase in plasma phytosterols [14]. This increase appears to be related to statin dose [15,16,24]. Although phytosterolemia, as recessive genetic disease related to deficiency of ABCG5/G8 carriers, is very rare, mild to moderate increases in phytosterolemia may be associated with increased cardiovascular risk [4,25], although this topic is still controversial [26]. The greatest contribution of our study was to show that the use of fibers in patients on highly effective lipid-lowering therapy, in order to reduce LDL-C > 50% and attain guideline goals, can prevent the increase in plant sterols plasma levels. Furthermore, among patients receiving therapy with simvastatin and ezetimibe, the use of fibers produced significant decrease in phytosterolemia. These findings seem of importance, because they support evidence for supplementation of fibers being a safe strategy when added to the most effective lipid-lowering strategies, reducing the absorption of phytosterols. These aspects seem yet more relevant for subjects bearing common polymorphisms of the NPC1L1 or ABCG5/G8 genes. Genetic variation in these genes were reported and can increase the absorption of sterols or decrease sterol extrusion to the intestinal lumen, which are associated with increased levels of phytosterolemia [27].

Other benefits of fiber supplementation observed in our patients were weight loss and the achievement of lower body mass index. The reduction in total energy intake may have contributed to these results, however, differences between groups were only observed in patients receiving fiber supplementation. Our findings are in agreement with previous studies showing inverse relationship between fiber intake and weight loss [28,29].

Another interesting finding of the study was the mild reduction in blood glucose in subjects receiving fiber supplementation. Previous studies have demonstrated reduction in fasting glucose, postprandial and glycated hemoglobin levels associated with soluble fiber intake [30-33]. Recently, two meta-analyses have shown a slight increase in the rates of new-onset diabetes mellitus in patients treated with statins [34,35]. Another contribution of fiber intake is the potential benefit for reduction in new cases of diabetes, attributed to statin therapy.

### Study strenghts and limitations

According to a recent meta-analysis of statin trials [36], lipid-lowering therapy that promotes greater reductions in LDL-cholesterol produces definite further reductions in the incidence of cardiovascular events. Therefore, our study tested the benefit of fiber intake in this scenario.

It is possible that the lipid effects of soluble fiber have been masked by the highly effective treatment used in our study. Furthermore, fiber effects on anthropometric parameters could be more pronounced in subjects with obesity and/or diabetes, and when fiber is consumed at longer periods.

## Conclusions

Soluble fiber intake in patients receiving effective strategies with lipid-lowering drugs seems important for the achievement of lower phytosterolemia (synergistic action with ezetimibe), and is associated with weight loss, and lower levels of plasma glucose.

SCR carried out the clinical protocol, performed statistical analysis and drafted the manuscript. FAF conceived of the study, participated in its design and coordination, performed statistical analysis and drafted the manuscript. SHK carried out the assessment of plasma sterols. FTM carried out the clinical protocol. TH performed statistical analysis; NCB and RAM standardized the assay of plasma sterols. VMR and FCS carried out the assays of plasma sterols. MCI conceived of the study, participated in its design and coordination, performed statistical analysis and drafted the manuscript.

All authors read and approved the final manuscript.

## Competing interests

The authors declare that they have no competing interests.
